# Observation of magnetic domains in graphene magnetized by controlling temperature, strain and magnetic field

**DOI:** 10.1038/s41598-020-78262-w

**Published:** 2020-12-07

**Authors:** Mahsa Alimohammadian, Beheshteh Sohrabi

**Affiliations:** Department of Chemistry, Surface Chemistry Research Laboratory, Iran University of Science and Technology, Tehran, 16846-13114 Iran

**Keywords:** Chemistry, Materials science, Nanoscience and technology

## Abstract

Since the production of ferromagnetic graphene as an extremely important matter in spintronics has made a revolution in future technology, a great deal of efforts has recently been done to reach a simple and cost-effective method. Up to now, controlling the magnetic properties at extremely low temperature have been investigated only by adding and removing atoms in graphene lattice. In this regard, the effect of strain on the magnetic and electronic properties of graphene has been probed. Here, the ferromagnetic properties are what have been created by strain, magnetic field, and temperature along with observation of the parallel magnetic domains in ferromagnetic graphene for the first time as a great achievement. In this way, we have represented the following: First, introducing three novel methods based on temperature, magnetic field, and strain for producing ferromagnetic graphene; Second, obtaining ferromagnetic graphene at room temperature by significant magnetization saturation in mass-scale; Third, probing the electronic systems and vibrational modes by Raman and IR spectroscopy; Fourth, introducing stacking and aggregation as two types of gathering process for graphene sheets; Fifth, comparing the results with leidenfrost effect-based method which the temperature, magnetic fields, and strain are simultaneously applied to graphene flakes (our previous work).

## Introduction

Graphene, as a 2D material^1^, is an excellent candidate for replacing many conventional materials in various applications due to remarkable electrical^1,2^, thermal^3^, mechanical^4^, and optical^5,6^properties. In the honeycomb structure of graphene, the presence of one free electron per atom is responsible for high electron mobility^1,7^and the carriers transport is described by Dirac equation^2^. Moreover, other electrical properties such as room-temperature quantum Hall effects^8^, unique band structure, and ambipolar electric field effects^1,2^, covers the electronic application.

Apart from electronics, graphene is also attractive material in spintronics, where in addition to charge, the spin of electrons is considered^9^. Indeed, graphene is suitable for spin logic devices due to its properties such as room-temperature spin transport with long spin-diffusion lengths^9,10^. Although the magnetic properties do not naturally exist in graphene, it is valuable in spintronics. Recently, the magnetic property is created in graphene by manipulating electronic systems. Interestingly, magnetic domains structure directly effects on magnetic properties. Generally, ferromagnetic materials are composed of one magnetic domain, with all dipoles aligned in the same direction which is a consequence of a strong driving force for parallel alignment caused by exchange energy^11^ . This energy is a quantum mechanical effect which tends to align electron spins, and in consequence their magnetic dipole moments, simultaneously. Noticeably, magnetic domains formation would certainly minimize the exchange energy; hence, it reduces total magnetic energy of ferromagnetic materials formation and raises their stability. By the same token, production of these domains in graphene during magnetization can thus make a revolution in future technology. Essentially, magnetism in graphene can be created by many methods such as functionalization, doping, and adding atoms. In fact, the symmetry of the electronic structure is locally broken around the new bonds and the magnetic moments are created in this area^12–30^. Additionally, vacancy and edge defects, Introduced as another method in magnetization. By removing one carbon atom and rearrangement of others in vacancy defect, the magnetic moment is created due to remaining a dangling bond which is theoretically estimated 1 μ_B_. Experimentally, vacancy defects are created by ionic bombarded and reduction of graphene oxide^13,31–41^. Among these methods, the highest magnetization is occurred in the doping methods by sulfur^25–27^ and nitrogen^23,24^ but at extremely low temperatures.

Observation of the pseudo-magnetic field in strained graphene without damaging the lattice structure is the huge gap in the magnetization process^42–44^. Indeed, the creation of the pseudo-magnetic field in highly strained nanobubbles of graphene is one of the extreme approaches in graphene magnetism^42^**.** In our previous study, Leidenfrost effect-based (LFE) method for preparation ferromagnetic graphene are introduced^45^**.** Evaporating graphene droplets under high temperature and external magnetic field leads to magnetism in graphene. In this method, temperature, magnetic field, and strain is simultaneously applied to graphene flakes and magnetization occurred. Here, all these parameters are considered separately and determined their contributions.

Graphene properties are extremely influenced by the preparation methods^46,47^. After first isolation of graphene by Scotch tape^1^, many methods are proposed to produce graphene, from exfoliation^48–50^ of graphite to growing graphene on the substrate by nucleation of carbon atoms^51^. On the other hand, most mechanical exfoliation methods^49^ such as the Liquid-phase exfoliation^52,53^ produce high-quality graphene flakes in mass-scale. Therefore, in this study, the graphene flakes are obtained by the Liquid-phase exfoliation method by using of ethanol solution^54^**.**

## Method

### Graphene preparation

**Figure 1:**
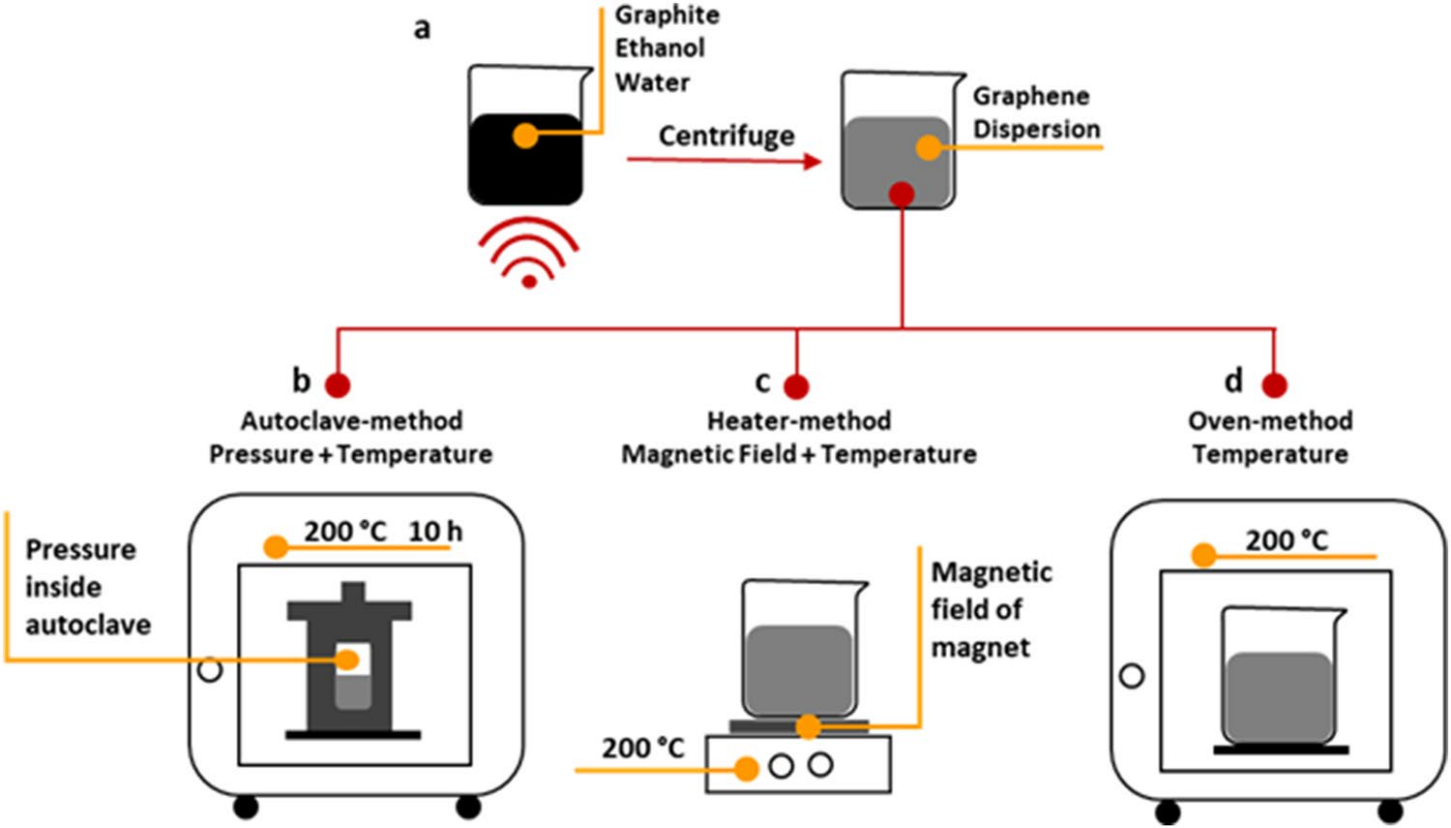
Dispersion of graphene and three different methods for investigating the effect of temperature, pressure, and magnetic field on the magnetization of graphene. (**a**) Dispersion of graphene dispersion by the liquid-phase exfoliation method. (**b**) Appling temperature and pressure by the Autoclave-method. (**c**) Appling magnetic field and temperature by the Heater-method. (**d**) Appling temperature by the Oven-method.

Graphene suspensions were prepared by the Liquid-Phase exfoliation method^52,54^**.** To follow the instruction, pristine graphite was added to the ethanol solution in which the graphite concentration was 5 mg/ml and the ethanol/water ratio was 20:80. Then, sonication was performed for 30 min at high power and was subsequently centrifuged at 3000 and 4000 rpm (just for LFE) for 10 min, to eliminate larger flakes (Fig. 1a). At these speeds, according to the Beer-Lambert equation with absorbance coefficient ~ 3182 l/g m (660 nm)^54^, the concentration of graphene was calculated to 2.9 and 2.1 mM, respectively. To consideration the role of temperature, pressure, and magnetic field in the magnetization process, three different methods were recommended, depicted in Fig. 1.

### Autoclave-method

As shown in Fig. 1b, the graphene suspension was poured in autoclave vessel and heated 10 h at 200 °C. In 10 h, the graphene flakes were aggregated and deposited on the bottom of vessels. Then, aggregated graphene was collected and dried. In this method, the graphene flakes were exposed to high pressure inside of autoclave and high temperature.

### Heater-method

As shown in Fig. 1c, the resultant suspension was placed on the Heater-stirrer at 200 °C until the solution was evaporated and graphene flakes remained. In this case, the magnetic field of magnet in heater-stirrer is considered as an external magnetic field source. The magnetic field, measured by a magnetic field sensor, was ~ 1 μT. In this process, both magnetic field and temperature were applied to graphene flakes.

### Ove-method

As shown in Fig. 1d, suspension of graphene was placed in the oven at 200 °C until the solution was evaporated and graphene flakes totally dried. The deposition was collected and used for further characterization. Here, the graphene flakes were exposed to just temperature.

### LFE-method

Aluminum plates were used and placed on the Heater-stirrer. The resultant suspension was dropped on the aluminum hot surface (300 °C) and after drying the ferromagnetic graphene particles were collected. Here, the droplets were rotated and evaporated base on the Leidenfrost effect. In this method, the source of the magnetic field and temperature were Heater-stirrer and the source of pressure was the pressure inside the droplets. In fact, all parameters were simultaneously applied to graphene flakes^45^.

## Results

**Figure 2:**
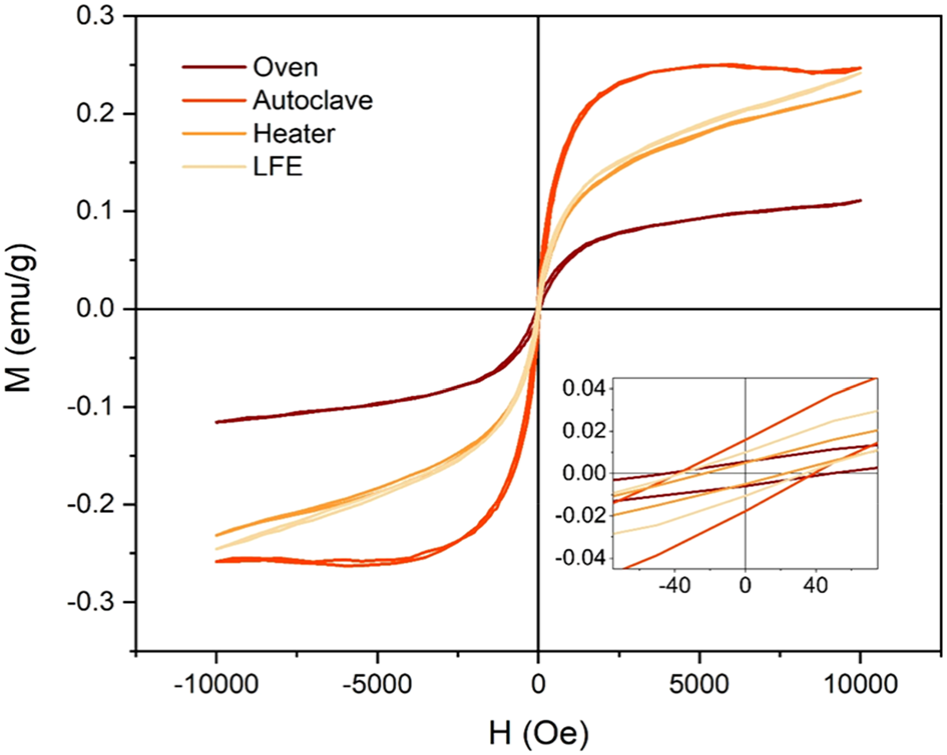
VSM diagram of FGPs to determine the contribution of temperature, pressure, and magnetic field in magnetism. The hysteresis loop of all samples and the zoom part of diagram − 90 < H < 90 (Oe) and − 0.045 < M < 0.045 (emu/g) in the inset.

**Figure 3:**
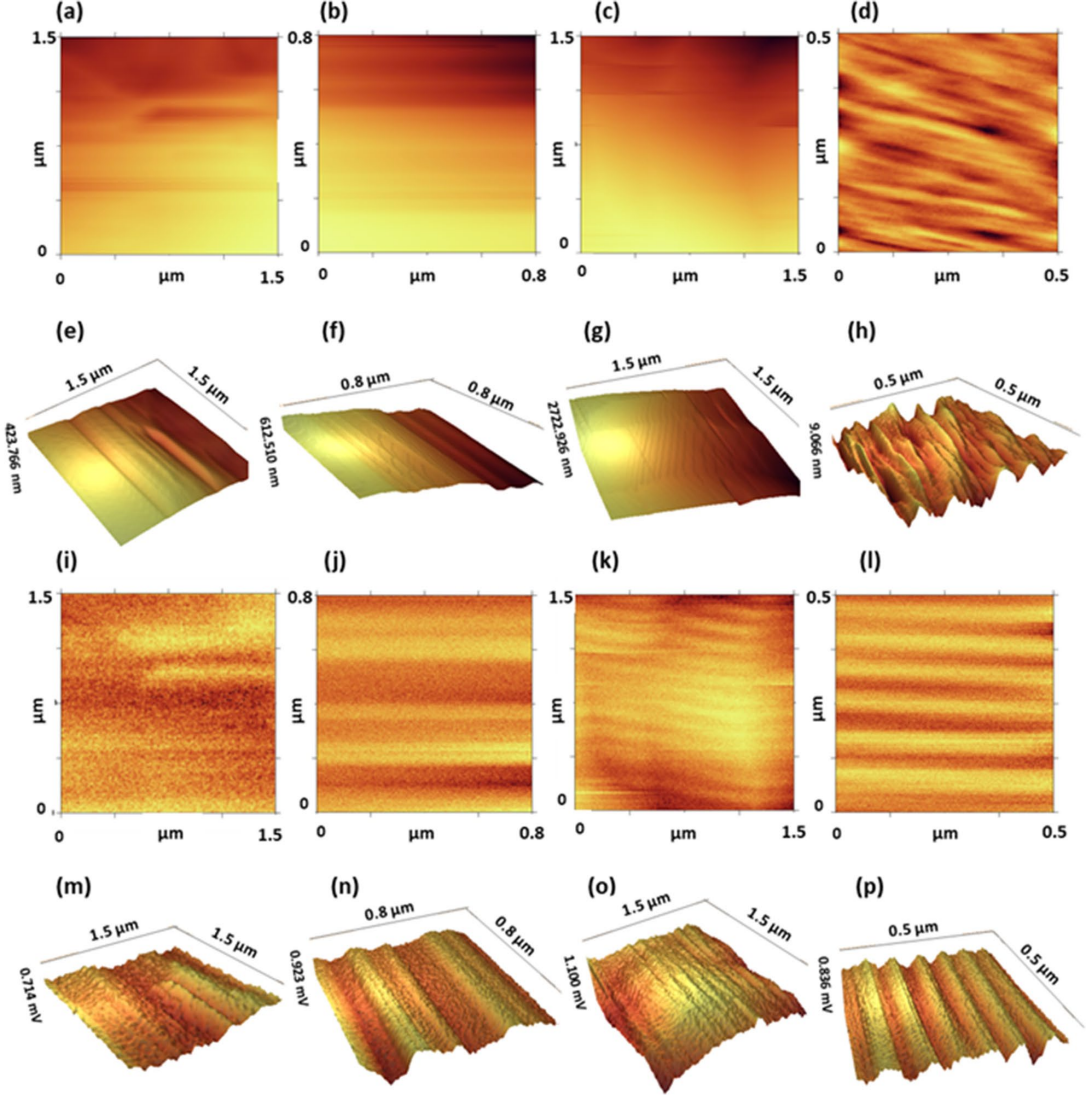
MFM images of all samples in Oven-, Autoclave-, Heater-, and LFE-method (left–right). (**a–d)** 2D topography images. (**e–h**) 3D topography images. (**i–l**) 2D amplitude images. (**m–p**) 3D amplitude images of FGPs in Oven-, Autoclave-, Heater-, and LFE-method, respectively (left–right).

**Figure 4:**
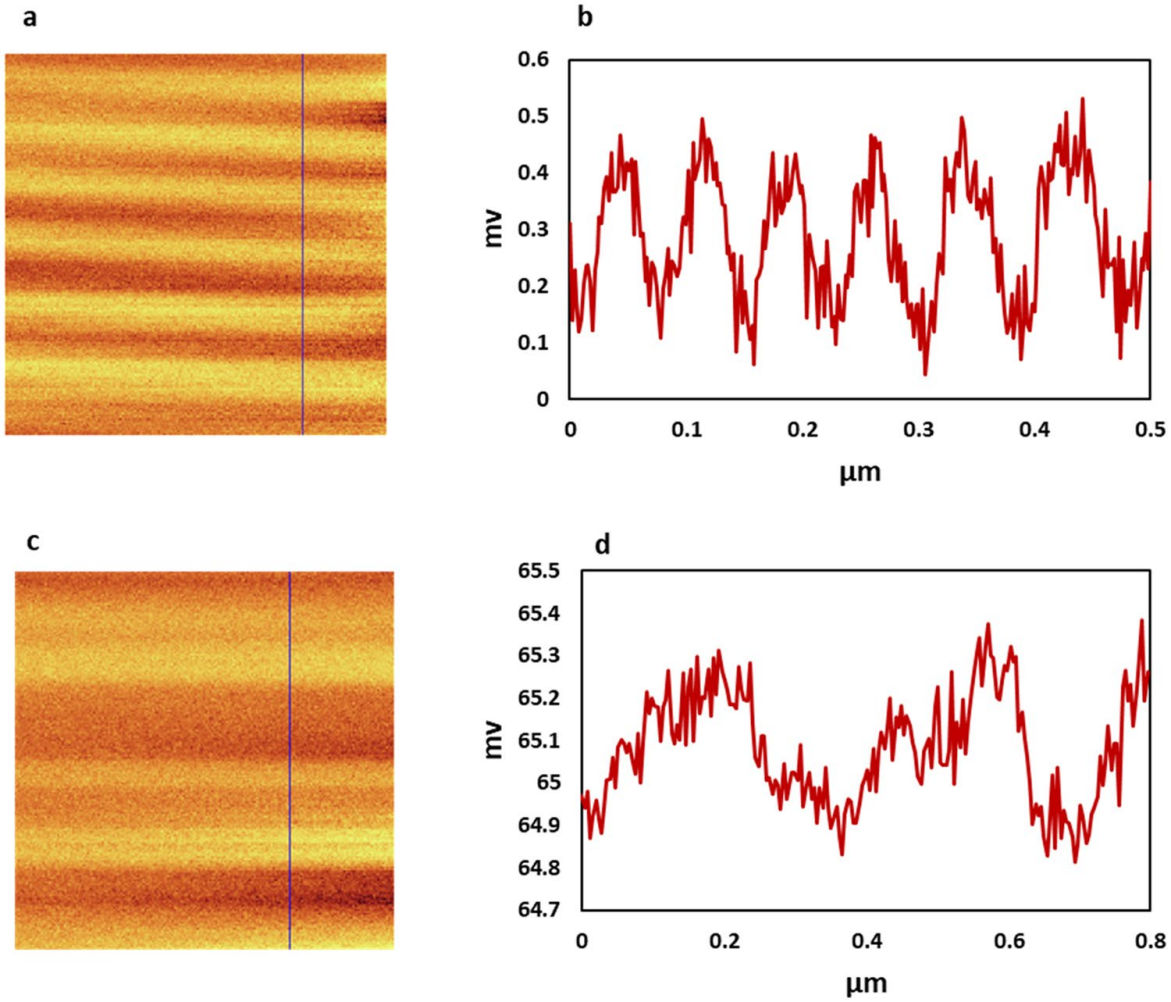
Magnetic domains of LFE- and Autoclave-samples and their domain size. Amplitude images and profile of blue line (**a,b**) LFE-method. (**c,d**) Autoclave-method.

**Figure 5:**
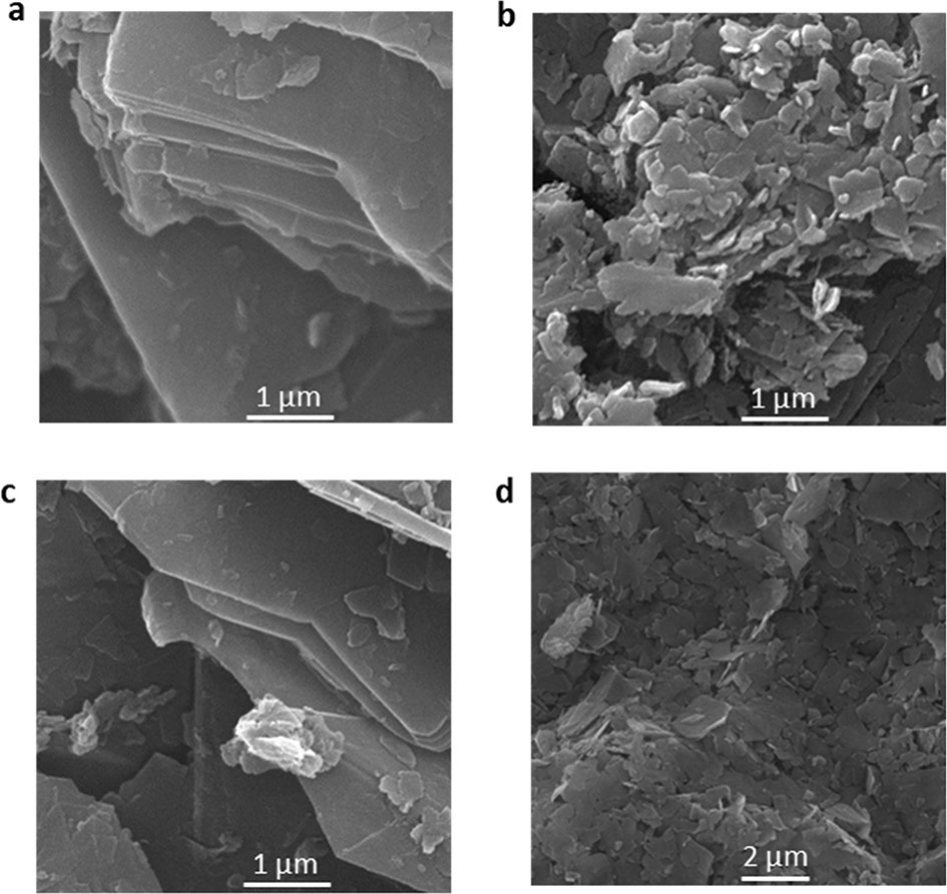
SEM images of FGPs. (**a–d**) the Oven, Autoclave, Heater, and LFE, respectively. The difference between stacking and aggregation process is obvious in the images. (**a,c**) Stacking. (**b,d**) Aggregation.

**Figure 6:**
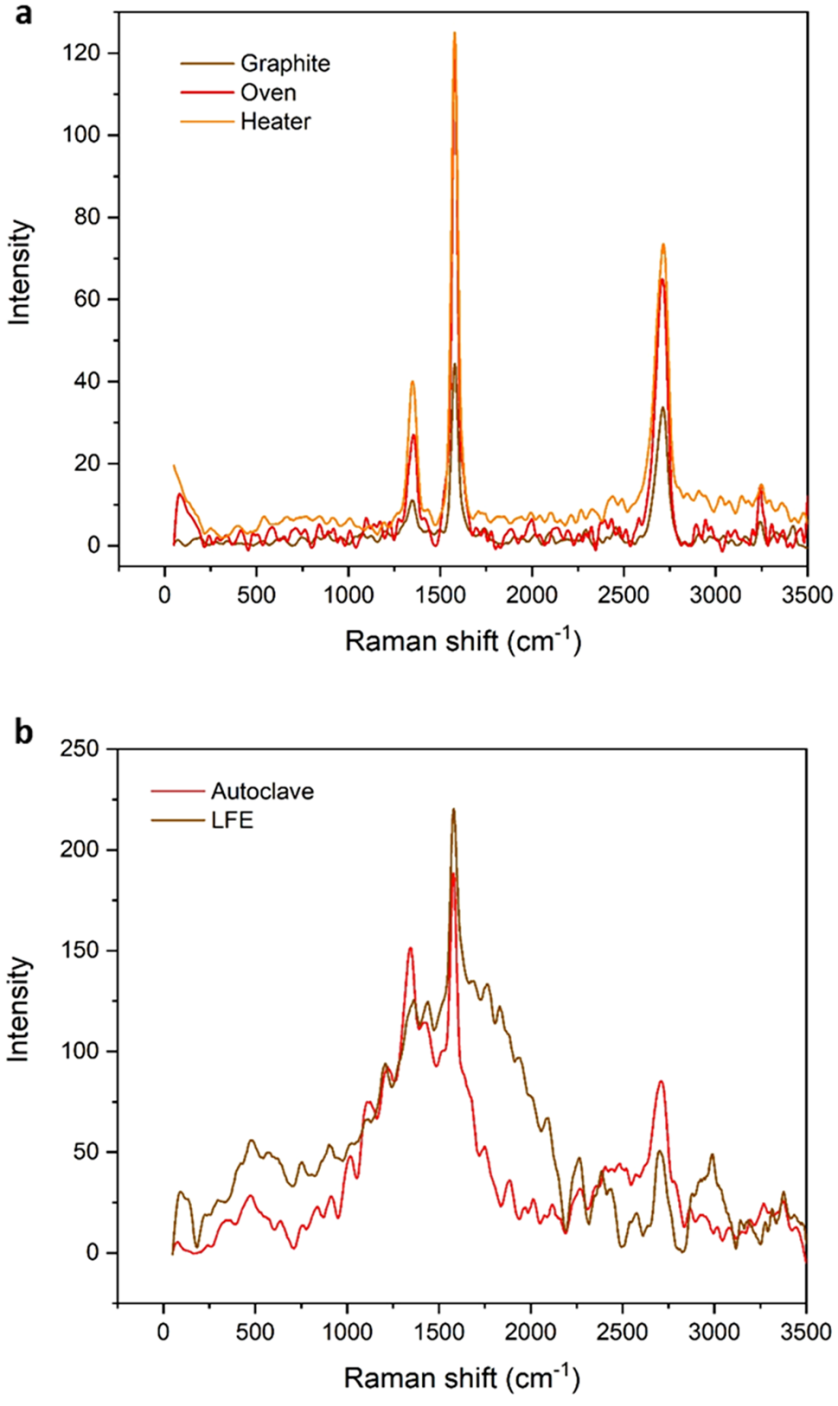
Vibrational modes of FGPs samples in Raman spectra. (**a**) Raman spectra of Graphite, Oven- , and Heater-method**.** (**b**) Raman spectra of Autoclave- and LFE-method.

As mentioned above, the strain is presented as an important factor to control and manipulating the electronic system and the magnetic properties of graphene^42–44,55^**.** Apart from strain, temperature and the magnetic field are significant parameters to manipulating the electronic and magnetic properties of graphene which is studied here. In all the methods, ferromagnetic graphene powders (FGPs) was created (Fig. 2) due to the change of electronic system and lattice structure of graphene. Also, the parallel magnetic domains are observed (Figs. 3 and 4) which can be related to self-arrangement behavior of electrons. In addition, the gathering process of graphene sheets can be directly affected the electronic systems and vibrational modes. Gathering of graphene flakes can be classified into two forms, stacking and aggregating. In the stacking process, the sheets can be gathered in a normal pattern (AB and ABC) but in the aggregation process, the sheets can be twisting and do not follow the normal pattern. It is expected that the gathering process is strongly related to the time of the process. In Oven- and Heater-methods, the gathering is performed slowly and sheets can be stacked in the normal pattern. Whereas, in LFE- and Autoclave-methods, the gathering process is performed rapidly and the sheets do not have time to stack in normal patterns, therefore the aggregation process happens. In Fig. 5, the difference between both aggregation processes are shown in the scanning electron microscopy (SEM). The electronic manipulation is detected by Raman spectroscopy (Fig. 6).

### Ferromagnetic graphene

Ferromagnetic behavior of graphene powders is investigated by vibrating sample magnetometer (VSM) and the results are shown in Fig. 2. Due to the natural defects (I_D_/I_G_ ~ 0.25) and negligible impurities such as Ni, Co, Fe, and Mn, the weak ferromagnetic behavior is observed in pristine graphite (Supplementary Fig. S1 and Table S1). As shown in Fig. 2, the magnetization saturation (Ms) of FGPs is different in the methods. In FGPs, ~ 0.08 emu/g as Ms is generated by temperature in Oven-method, ~ 0.24 emu/g is generated by temperature and pressure in Autoclave-method, and ~ 0.16 emu/g is generated by temperature and the magnetic field in Heater-method. Clearly, the contribution of temperature, pressure, and magnetic field in the magnetization of graphene is ~ 0.08, 0.16, and 0.08 emu/g, respectively. Because of the better comparison in magnetic domains, the 4000 rpm sample of LFE is used in this study (Ms ~ 0.16 is near to other FGPs). Therefore, for comparison of Ms, the Ms of 3000 rpm LFE-sample is reported ~ 0.4 emu/g, indicated the simultaneous effect of all parameters^45^**.**

Similar to LFE-method, the slope created in the VSM diagram of Heater-method in high magnetic fields, related to the non-uniformity of particles. In Heater-method, the non-uniform conditions are felt by flakes because of gradual removal of the solution, non-uniform of the magnetic field, and non-uniform heat flow. In LFE-method, the slope created is related to the different behavior of droplets^45^**.** Because the temperature and pressure are applied in all directions, all flakes can be felt the same conditions in Oven- and Autoclave-method. The details of the hysteresis loop were summarized in Supplementary Table S2 and are in the inset of Fig. 2.

### Magnetic domains

Magnetic force microscopy (MFM) is used to observe the magnetic domains. This technique is similar to atomic force microscopy (AFM), based on cantilever oscillations. The surface of materials is probed by the magnetic tip in different distances. Each line is scanned twice, in one of them, the tip moves near the surface to collect the topography data and in another one the tip moves far from the surface to collect magnetic data^56^. In Fig. 3, 2D and 3D form of topography and amplitude images for FGPs are presented. Phase images are similar to amplitude, which are presented in Supplementary Fig. S2. All data were collected by a silicon magnetic tip coated by Co and Cr. Because of the unstable small flakes on the powders, moved by the tip, a lot of noises are created and disrupted images. Hence, the disrupted part is cropped that is why the scale of images are different. In the comparison of the topography and amplitude images, the clear magnetic lines are observed in amplitude at flight modes, which indicate parallel magnetic domains. However, the magnetic domains are observed in all samples, LFE- and Autoclave-sample have particular pattern and the grain size of them are ~ 0.1 and 0.4 μm, respectively (Fig. 4).

### Raman and IR spectroscopy

Raman and IR spectrum complement each other and show an excellent map of active vibrational modes. By means of these techniques, a lot of information is obtained about the electronic systems and the lattice vibrational modes of graphene at Brillouin Zone (BZ). Raman spectroscopy is widely considered as a key diagnostic tool for symmetric vibrational modes, whereas IR is used for recognizing the asymmetric modes. Although most vibrational modes are active, some of the modes are silent. Generally, in Raman spectroscopy, the laser light is used to excitation and Raman scattering is detected. In addition, the electronic band structures can involve in the resonance process such as double resonance (DR) and triple resonance (TR) Raman process. For example, the phonon modes far from the center of BZ are activated by the DR Raman process. Whereas, at Γ point the phonon is usually activated by first-order Raman process^57–60^**.**

Lattice structure, the lattice vibrations, the electronic systems, and their changes can be investigated by Raman spectroscopy. Therefore, each parameter that affects the electronic systems and phonon modes of graphene can be detected and probed by Raman spectroscopy^57^. Raman spectra of all FGPs and graphite are shown in Fig. 6. Also, all effective parameters in these Raman spectra such as the number of layers, stacking order, twisting, and also the external perturbation such as strain, magnetic field, and temperature are discussed below. The wavelength of the laser used in this study is 532 nm and its spot size is 0.72 μm. Indeed, the area of the sample is covered by laser light is 0.72 μm, in each measurement. Many flakes can exist in this area which is different at the number of layers, stacking order, twisting, and electronic systems.

Interpretation of the vibrational mode is based on the structural symmetry and group theory. The highest symmetry is D_6h_, which is belonged to one-layer graphene and graphite, at the Γ point. The irreducible representation of them are Γ_Graphene_ = A_2u_ + B_2g_ + E_1u_ + E_2g_ and Γ_Graphite_ = 2(A_2u_ + B_2g_ + E_1u_ + E_2g_). Three optical modes in graphene are E_2g_ (Raman active) and B_2g_ (silent). In graphite, nine optical modes exist containing three IR active, five Raman active, and one silent (B_2g_) modes. IR active modes are a doubly degenerate E_1u_ appear in ~ 1588 cm^−1^, generated from asymmetric in-plane vibrational mode and one asymmetric out-of-plane mode, A_2u_, located in ~ 868 cm^−1^ ^57,61^. In Supplementary Fig. S3, the IR spectrum for all samples is presented and E_1u_ and A_2u_ modes are observed in most samples. Also, these two peaks are too weak in graphite and LFE-sample. For graphite and LFE-sample, E_1u_ peak is located at ~ 1525 cm^−1^ and in the other samples broadening and redshift of E_1u_ peak is observed.

By increasing the number of layers, the symmetry is reduced and subsequently, the modes are changed. For example, N-layer graphene (NLG) symmetry with the even and odd layers is D_3d_ and D_3h_ at Γ point, respectively. Also, the symmetry information along the Γ–K direction is different and their phonons are activated by DR Raman process. For example, the symmetry of monolayer and NLG (even) at M point is D_2h_ and C_2h_, respectively^58,62^. Generally, changes in the electronic system and the lattice structure can create new modes, and even the combination and splitting of some modes can appear the complex modes in the Raman spectrum.

Basically, three characteristic peaks exist in graphene Raman spectra. One of the main peaks in Raman spectra is generated from symmetric in-plane vibrational mode, located in ~ 1580 cm^−1^. These doubly degenerate, E_2g_, is called G mode and its position is sensitive to external perturbations, such as defects, doping, strain, and temperature. The two other peaks appear in ~ 2670 and 1200 cm^−1^, called 2D and D, respectively^57^.

### Ultralow-frequency modes

Ultralow-frequency modes are directly related to interlayer vibrations. In graphite, the out-of-plane and in-plane interlayer vibrations, layer-breathing (LB) and shear (C) modes, are related with B_2g_ (~ 128 cm^−1^) and E_2g_ (~ 43.5 cm^−1^) modes, respectively. As another example in AB-2LG, C and LB modes are located at ~ 31 cm^−1^ (E_g_) and ~ 90 cm^−1^ (A_1g_), respectively. In addition, C mode in AB-3LG and ABC-3LG are located at ~ 33 cm^−1^ and ~ 19 cm^−1^, respectively. Generally, interlayer vibrations depend on twisting, stacking order, and the number of layer^57,61,63–68^. In Fig. 4a, the Raman spectra of graphite, Heater-, and Oven-sample are shown and no special modes are observed between 0 and 1000 cm^−1^, however, many modes are observed in Fig. 4b for LFE- and Autoclave-sample. These differences are related to the aggregation and stacking process.

### D mode

D mode requires defects for activation. This mode is used to characterize defects in graphene. Generally, the I_D_/I_G_ is used to estimate the amount of defects in graphene flakes^57^. Only edge defects can be created in the samples, related to the sonication process in the dispersion of graphene. Because of using the same dispersion method in all samples, it is expected that the edge defects are the same and I_D_/I_G_ are equal (Supplementary Table S3) but in aggregation process the number of edges, exposed to the laser light are more than the stacking. Moreover, the complexity of D mode in Supplementary Fig. S4b related to combination and creation new modes.

### G mode

G mode is originated from a first-order Raman scattering process and related to E_2g_ mode at Γ point. In graphene and graphite, G mode is located in ~ 1582 cm^−1^ and is sensitive to external magnetic fields, strain, and temperature. According to published studies, discrete Landau levels are created in graphene by perpendicular magnetic fields. In addition, the energy and filling factor of Landau levels depend on the strength of the external magnetic field. Electron transition between these levels and resonance process with optical phonons are known as magneto-excitons and magneto phonon resonances, respectively. These are led to new optically modes, detected by Raman spectra. For example, when one-layer graphene is exposed to the perpendicular magnetic field, the symmetry representation of allowed transitions are A_1_, A_2_, and E_2_ which the E_2_ and E_2g_ (origin of G peak) can interact. Therefore, the position and the shape of the G peak are changed and led to broadening and splitting^57,69–72^. Broadening of G mode in Heater-method can be related to the new modes such as E_2_ (Supplementary Fig. S4c).

Changing the interatomic distance in crystal lattice under stain and stress lead to manipulating electronic systems. Recently, the strain effect on electronic systems is considered and remarkable results such as shifting the Dirac cones, opening band gap, shifting and splitting Raman modes, and inducing strong pseudo-magnetic field is reported. As an example, in graphene, shifting the vibrational frequency and splitting the G mode (G^+^ and G^−^) is observed under stress. In addition, the G mode are sensitive to temperature^42,57,73–80^.

### 2D mode

2D (~ 2700 cm^−1^) is activated by the resonance process. The component of the 2D peak dependent on electronic bands and laser wavelengths. Here, the laser wavelength is the same in all measurements. Due to several electronic bands are in multilayer graphene, the many 2D components can be expected. Because of these features, the 2D peak is used to determine the number of layers. In addition, the stacking order also can be strongly effected on electronic bands and change the spectral profile of the 2D peak. Therefore, the complexity of 2D LFE-sample and Autoclave-sample compared to other samples can be related to more manipulation of electronic systems (Fig. 6b). In addition, 2D peak is sensitive to temperature and strain. Moreover, in the presence of strain, deformation of Dirac cones is led to splitting 2D modes into 2D^+^, and 2D^−^^57,66,76,81–84^.

## Conclusion

Here, three different methods are reported for the production of FGPs. Characterize of FGPs are performed by VSM, MFM, Raman, and IR spectroscopy. Magnetism is determined and magnetic domains are observed. To discussion about the results, the electronic systems and phonons are investigated. Magnetization process is based on manipulating the electronic system under temperature, external magnetic field, strain, and is also related to the gathering process. In conclusion, all three parameters can manipulate the electronic systems and control the magnetic properties of graphene powders. The best parallel magnetic domains are observed in Autoclave- and LFE-samples.

### Materials and characterization

Pristine graphite is purchased from Merck Company and its impurities are determined by inductively coupled plasma (ICPS-7000, SHIMADZU). Ethanol solution is prepared by pure ethanol (Merck) and deionized water. After dispersion, elimination of larger flakes is occurred by centrifuge (Hettich EBA20). To prepared ferromagnetic graphene Heater-stirrer (Heidolph, MR Hei-standard) and oven (Memmert) are used. For investigating magnetization of graphene, Vibrating Sample Magnetometer (VSM, Kavir, Iran) is used. Natural defects of graphite and manipulation of electronic structure are investigated by Raman spectroscopy (HORRIBA). UV–Vis (mini 1240) and FTIR (8400S) spectrophotometer analyzes are performed by SHIMADZU instruments for investigation graphene concentration and vibrational modes, respectively. Magnetic field sensor (Narba, NBM550, and German) is used to measure magnetic fields of Heater-stirrer.

## Supplementary information

Supplementary Information.
