# Supplementary material for: Observation of magnetic domains in graphene magnetized by controlling temperature, strain and magnetic field
Source: Sci Rep. 2020 Dec 7;10:21325. doi: 10.1038/s41598-020-78262-w (PMC7721724; doi:10.1038/s41598-020-78262-w)
Supplement: Supplementary file 1 — Supplementary Information. [file 41598_2020_78262_MOESM1_ESM.docx]

Supplementary Information for

**Observation of magnetic domains in graphene magnetized by controlling temperature, strain and magnetic field**

Mahsa Alimohammadian ^a^, Beheshteh Sohrabi *^, a^

*^a^ Department of Chemistry, Surface Chemistry Research Laboratory, Iran University of Science and Technology, 16846-13114, Tehran, Iran*

**FIG. S1 | Ferromagnetic behavior of pristine graphite.** Weak ferromagnetic behavior is originated from Impurities and natural defects. Impurities are investigated by ICP spectroscopy and its data are summarized in Supplementary Table. S1. Natural defects are determined in Raman spectra of graphite (I_D_/I_G_=0.25).


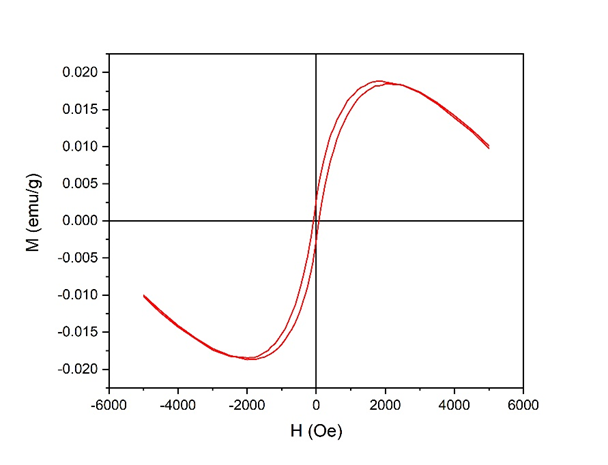


**FIG. S2 | Magnetic domains in LFE-, Heater- Autoclave, and Oven- samples. (a, c, e, g)** 2D phase images**. (b, d, f, h)** 3D phase images**.**


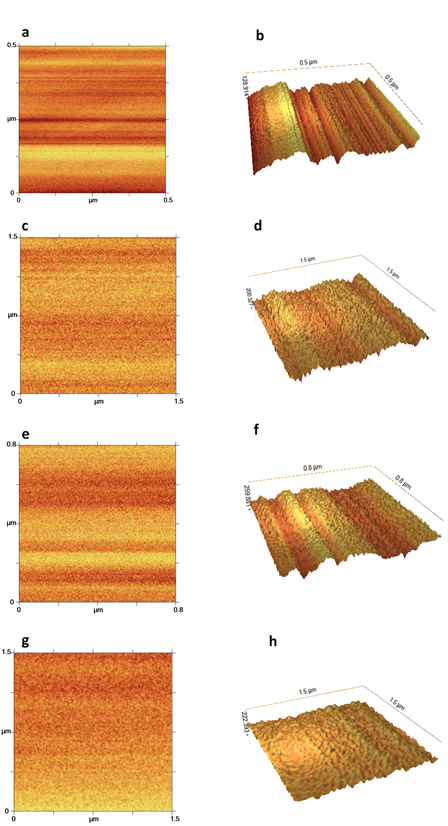


**FIG. S3 | IR spectrum of FGPs.** A_2u_ (~1017 cm^-1^) and E_1u_ (~1512 cm^-1^) mode are activated and summarized in Table. S3. Because of trapping water molecules in Oven-, Heater, and Autoclave- method, a wide peak appears ~ 3500 cm-1. IR active modes are a doubly degenerate E_1u_ appear in ~1588 cm^-1^ and A_2u_, located in ~ 868 cm^-1^.


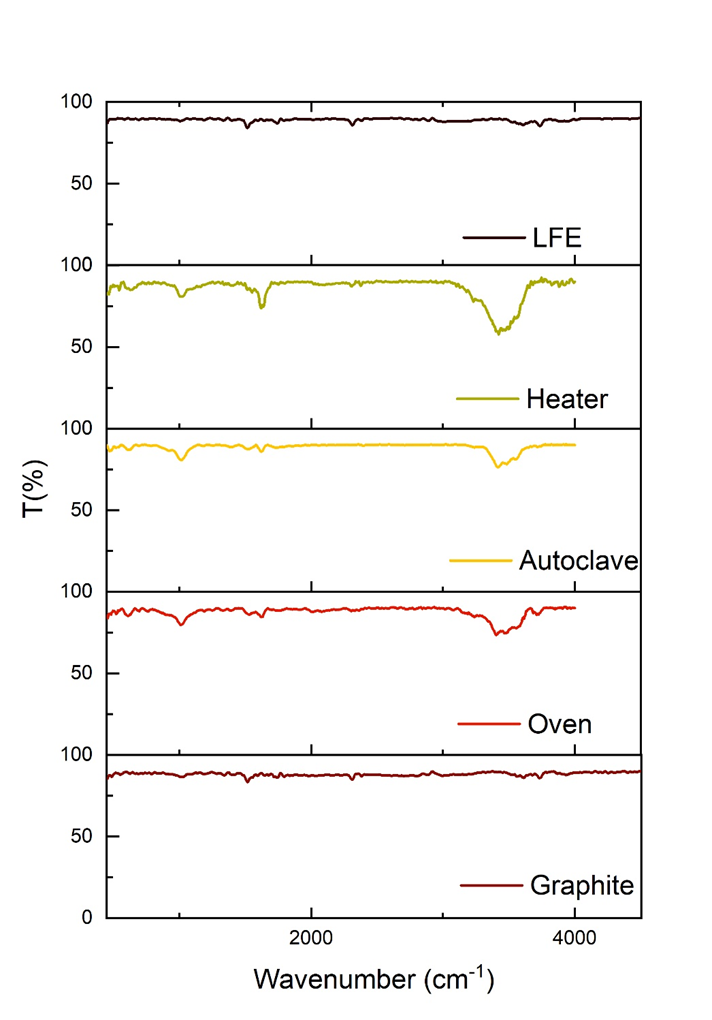


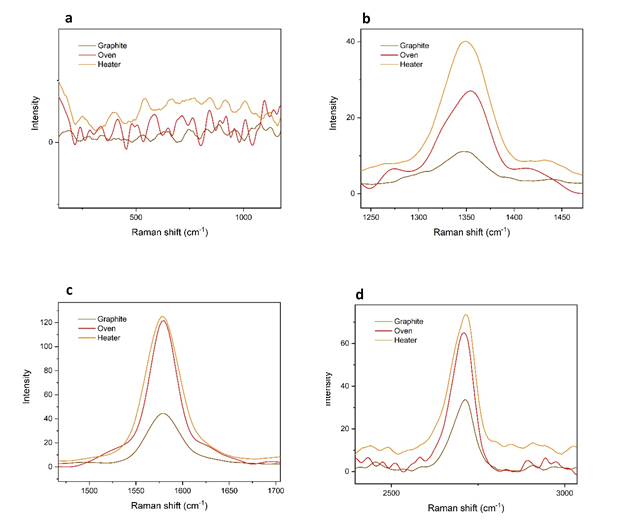


**FIG. S4 | Zoom in Raman peaks. (a-d)** ultralow-frequency, D mode, G mode, and 2D mode for Fig. 6a, respectively. The data are summarized in Table. S3. **(b-d),** The broadening of D, G, and 2D modes is obvious.

| **Table. S1 \| Impurities of pristine graphite.** | | | | |
| --- | --- | --- | --- | --- |
| element | Fe | Ni | Co | Mn |
| Concentration | >1ppm | >0.1 ppm | <0.1 ppm | <0.1 ppm |

| **Table. S2 \| Summarized the VSM data from Fig. 2.** | | | |
| --- | --- | --- | --- |
|  | Mr (emu/g) | Hc (Oe) | Ms (emu/g)  (~3488 Oe) |
| Oven-method | 0.005 | -45.33 | 0.08 |
| Autoclave-method | 0.015 | -38.22 | 0.24 |
| Heater-method | 0.005 | -23.11 | 0.16 |
| LFE | 0.009 | -36.89 | 0.16 |

| **Table. S3 \| Summarized wavenumber (cm^-1^) of Raman and IR modes.** | | | | | | | |
| --- | --- | --- | --- | --- | --- | --- | --- |
| Raman | | | | | IR | | |
|  | LB & C modes | D mode | G mode | 2D mode | I_D_/I_G_ | A_2u_ | E_1u_ |
| Graphite | --- | ~ 1347 | ~ 1579 | ~ 2713 | ~ 0.25 | ~ 1004 | ~ 1525 |
| Oven-method | --- | ~ 1354 | ~ 1579 | ~ 2709 | ~ 0.22 | ~ 1017 | ~ 1471 |
| Heater-method | --- | ~ 1349 | ~1579 | ~ 2714 | ~ 0.32 | ~ 1017 | ~ 1512 |
| Autoclave-method | Active | ~ 1343 | ~ 1580 | ~ 2708 | ~ 0.8 | ~ 1017 | ~ 1485 |
| LFE | Active | ~ 1362 | ~ 1580 | ~ 2702 | ~ 0.57 | ~ 1004 | ~ 1525 |
